# Comparative Effectiveness of Homoeopathic vs. Conventional Therapy in Usual Care of Atopic Eczema in Children: Long-Term Medical and Economic Outcomes

**DOI:** 10.1371/journal.pone.0054973

**Published:** 2013-01-31

**Authors:** Stephanie Roll, Thomas Reinhold, Daniel Pach, Benno Brinkhaus, Katja Icke, Doris Staab, Tanja Jäckel, Karl Wegscheider, Stefan N. Willich, Claudia M. Witt

**Affiliations:** 1 Institute for Social Medicine, Epidemiology, and Health Economics, Charité University Medical Centre, Berlin, Germany; 2 Department of Paediatric Pulmonology and Immunology, Charité University Medical Centre, Berlin, Germany; 3 Department of Medical Biometry and Epidemiology, University Medical Centre, Hamburg-Eppendorf, Germany; 4 Centre for Integrative Medicine, University of Maryland School of Medicine, Baltimore, Maryland, United States of America; Universidade Federal do Acre (Federal University of Acre), Brazil

## Abstract

**Background:**

One in five children visiting a homeopathic physician suffers from atopic eczema.

**Objectives:**

We aimed to examine the long-term effectiveness, safety and costs of homoeopathic vs. conventional treatment in usual medical care of children with atopic eczema.

**Methods:**

In this prospective multi-centre comparative observational non-randomized rater-blinded study, 135 children (48 homoeopathy, 87 conventional) with mild to moderate atopic eczema were included by their respective physicians. Depending on the specialisation of the physician, the primary treatment was either standard conventional treatment or individualized homeopathy as delivered in routine medical care. The main outcome was the SCORAD (SCORing Atopic Dermatitis) at 36 months by a blinded rater. Further outcomes included quality of life, conventional medicine consumption, safety and disease related costs at six, 12 and 36 months after baseline. A multilevel ANCOVA was used, with physician as random effect and the following fixed effects: age, gender, baseline value, severity score, social class and parents’ expectation.

**Results:**

The adjusted mean SCORAD showed no significant differences between the groups at 36 months (13.7 95% CI [7.9–19.5] vs. 14.9 [10.4–19.4], p = 0.741). The SCORAD response rates at 36 months were similar in both groups (33% response: homoeopathic 63.9% vs. conventional 64.5%, p = 0.94; 50% response: 52.0% vs. 52.3%, p = 0.974). Total costs were higher in the homoeopathic versus the conventional group (months 31–36 200.54 Euro [132.33–268.76] vs. 68.86 Euro [9.13–128.58], p = 0.005).

**Conclusions:**

Taking patient preferences into account, while being unable to rule out residual confounding, in this long-term observational study, the effects of homoeopathic treatment were not superior to conventional treatment for children with mild to moderate atopic eczema, but involved higher costs.

## Introduction

Comparative Effectiveness Research (CER) is a growing field in health care research; it has considerable potential to inform stakeholders on decision-making. Different definitions for CER have been published. In this paper we use the working definition as established by the Institute of Medicine (IOM) Committee, which defines CER as “the generation and synthesis of evidence that compares the benefits and harms of alternative methods to prevent, diagnose, treat, and monitor a clinical condition or to improve the delivery of care”. The purpose of CER is to assist consumers, clinicians, purchasers, and policy makers to make informed decisions that will improve health care at both the individual and population levels [1]. CER is especially valuable for disorders that are most common and most costly to society, have the highest morbidity rates and have a great degree of variation in the treatment of the disorder [2].

Atopic eczema is a chronic inflammatory skin disease associated with pruritus, which occurs predominantly in children [3], [4]. Atopic eczema, as well as other atopic diseases, has become even more prevalent in Western industrialized countries in recent years, affecting around 7 to 8% children aged 6 to 7 and 13 to 14 with current eczema symptoms, and up to 22 to 25% worldwide [4]. The nationwide population-based German Health Interview and Examination Survey for Children and Adolescents (KIGGS) found a life-time prevalence of 13% and a point prevalence of 7% for atopic eczema in children and adolescents [5].

Atopic eczema can impose great burden on both the child’s and their parents’ overall wellbeing and has relevant economic impact on both the individual and to society [6], [7] with estimated annual costs of 1.1 to 3.6 Billion Euro [8], [9] in Germany alone.

Complementary medicine is increasingly asked for in the treatment of atopic eczema, as well as other allergic conditions [10]–[12]. Homoeopathy, for example, is widely used in Germany for treating atopic eczema, although official guidelines do not recommend it. Data from a cohort study showed that one in five children who sought a homoeopathic medical doctor suffer from atopic eczema [13]. However, very little data on the efficacy or effectiveness of homoeopathy for eczema is available. A meta-analysis on homeopathy included nine dermatological studies, none of which looked specifically at atopic eczema [14]. A small randomized, placebo-controlled trial could not show a superior effect of individualized homeopathic treatment over placebo [15]. In addition, most trials focus on the short-term effects, however this yields little insight into the longer treatment options for this chronic condition.

A comparative effectiveness research study (ADEV study) was performed to examine the effectiveness, safety and cost of homoeopathic vs. conventional treatment in usual care of children with atopic eczema taking patient preferences into account [16].

Children were included and followed between January 2005 and October 2009. The primary endpoint of the study was a symptom score after six months (SCORAD; SCORing-Atopic-Dermatitis). Those results (including a follow-up after 12-months) have already been published [16]. After six and 12 months homoeopathic treatment was not superior to conventional treatment and higher costs were observed in the homoeopathic compared to the conventional group.

Information on the long-term effects is of great interest, especially for chronic conditions. Thus, the aim of the present analysis was to describe the effectiveness and the costs involved in the long-term follow-up after 36 months.

## Materials and Methods

### Study Design and Participants

**Figure 1:**
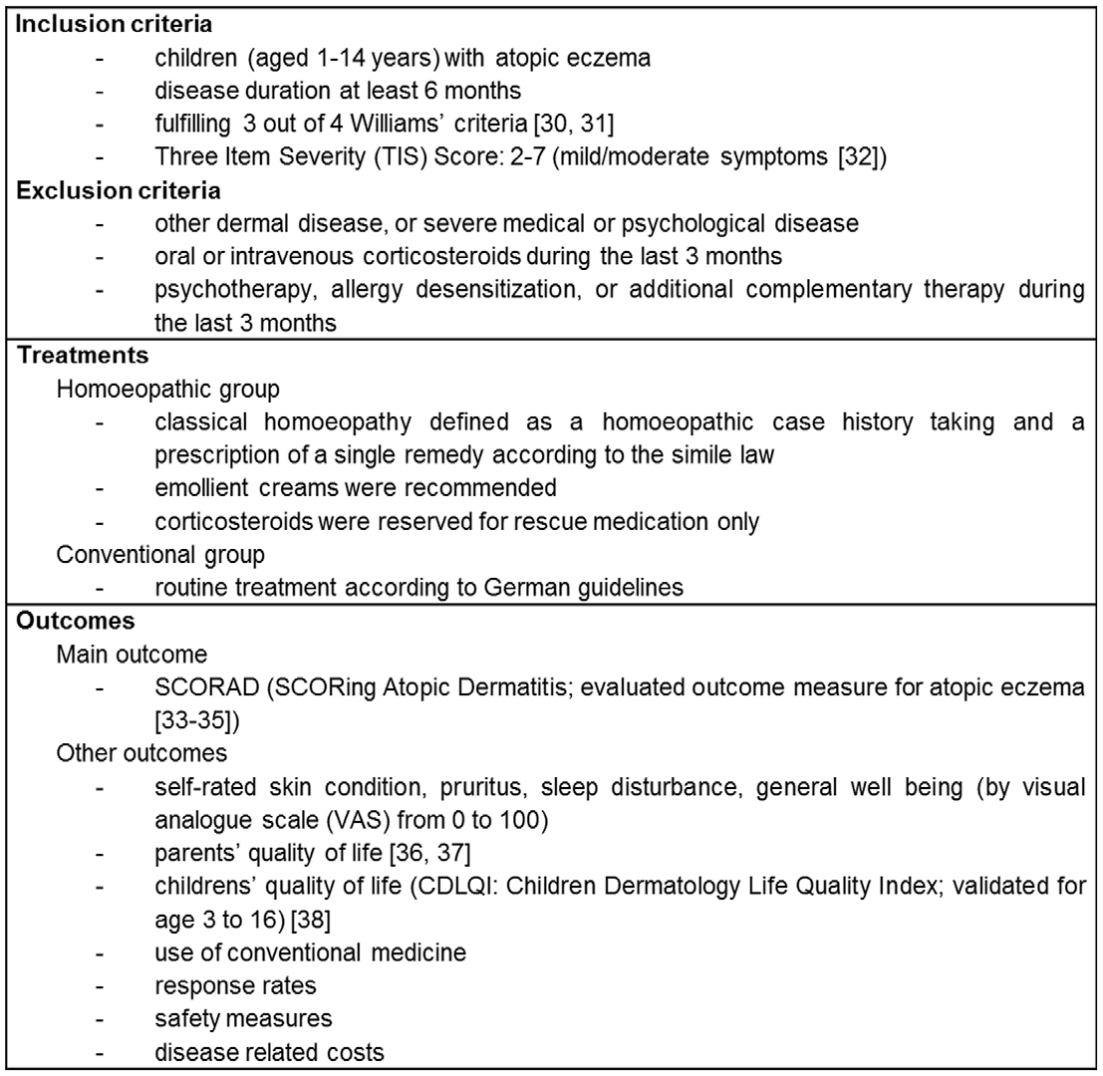
Description of inclusion and exclusion criteria, treatments and outcome measures.

Children were recruited from January 2005 to June 2006 in Berlin, Germany for this non-randomized prospective multicentre open comparative observational study. Data was collected up until October 2009 for the long-term follow-up, allowing a total observation period of 36 months per patient. Children and their parents were recruited at either homoeopathic or conventional doctors’ practices and had already made their own choice of therapy. Thus, the parents’ preference towards treatment of atopic eczema generated the groups to be compared. The recruitment of homoeopathic doctors was through the association of homoeopathic doctors in Berlin, while doctors for conventional treatment (paediatricians or dermatologists) were chosen from address lists or by recommendation. Further methods of this study have been described in detail previously [16]. Inclusion and exclusion criteria, intervention details and outcome measures are summarized in Fig. 1. The study was compliant with Good Epidemiological Practice (GEP) and applicable data-protection laws. Oral and written informed consent was obtained from the parent accompanying the child after verbal information about the study was provided by the physician. The signed consent form was sent to the central study center, and a copy was kept at the physician’s office. The study and the consent procedure were approved by the Ethics Committee of the Charité-Universitätsmedizin Berlin, Germany.

### Study Procedures

At baseline, a conventional case history, screening and recruitment took place at the physicians’ practice before the patients were enrolled in the study. Patients were then asked to complete a questionnaire on socio-demographic characteristics, outcome measures, and adverse events. The main outcome was the SCORAD (SCORing-Atopic-Dermatitis) score, which includes the rating of the extent and intensity of AD, as well as subjective items on pruritus and sleeplessness. To reduce bias, the SCORAD was centrally assessed by two specially trained staff members who were blinded of the treatment group. Patients were asked to respect the blinding of the rater. Each patient was assigned to one rater only for the whole study period to ensure intra-rater stability. After the central rating had taken place, patients could visit their respective physician and start with either the homoeopathic case history taking and the subsequent individualised treatment or the conventional treatment. The children’s physicians documented the treatment over a 12 months period. To reflect usual care, patients visited their physician whenever needed. At each visit, data was obtained by filling out questionnaires and ratings. For the final three-year follow-up, all patients that had attended at least one of the prior follow-up visits (at six or 12 months) were invited. After 36 months no follow-up data was retrieved from the physicians.

### Economic Analysis

The cost-comparison analysis was made from a societal perspective and was performed from a diagnosis specific view (only costs with direct relation to atopic eczema were considered). We summarized costs for 12 months before study onset (months −12 to months 0), and for the following 6 month periods after study onset: 1–6 months, 7–12 months and 31–36 months, where available. Data on resource consumption such as hospital stays, use of medication and days on sick leave were obtained from the patient questionnaires and diaries. Costs of days spent in hospitals were based on the appropriate dermatological German DRGs. The cost of medication was based on consumed units and package prices. If this data was not available, daily defined dosage [17] was multiplied by the number of days of intake. Outpatient visits were valued by multiplying the number of visits and the mean contact-costs depending on the physician’s profession (data provided by the Association of German Statutory Health Insurance Physicians). For the follow-up between months 31 and 36, the costs were discounted by 3% per year. For the first 12 months of the study, the costs incurred due to visits to the ADEV-study physician were directly extracted from the doctors’ documentation. For the long-term follow-up for the months 31 to 36, no physicians’ documentation was available. The mean cost values from the respective treatment group at months 7–12 were used for those patients who documented that they still visited their study physician. As a post-hoc sensitivity analysis we calculated the costs for these visits in months 31 to 36 for the treatment group to be 40% of the costs at months 7–12, as there is evidence for a reduction in costs in homoeopathic treatments over time [18].The cost for patients who had no further contact with their ADEV-study physician was valued at 0€.

Indirect costs were calculated by adopting the human capital approach. In cases of disease-related absence from work, the indirect costs were measured according to the parents’ income level.

### Statistics

Statistical analyses were based on the intention-to-treat (ITT) principle, including all patients with baseline values who received treatment and with assessed outcome using multilevel models (analysis of covariance (ANCOVA) or generalized estimating equations (GEE)). In these models, physicians were considered random effect and fixed effects were: baseline value (continuous), Three item severity (TIS) Score (continuous), social class (high, average, low), parents’ expectation of a good outcome (high, low), children’s age (continuous) and gender (male/female). Results are presented as adjusted mean or proportion with a standard error (SE) and/or 95% confidence interval (CI). All tests were exploratory and two-sided with a level of significance of 5%. Adverse events and intake of corticosteroids of different potency groups [19] were analyzed descriptively by frequencies, percentages and by Chi-squared or Fisher’s exact test (if feasible). As a sensitivity analysis, analysis was additionally performed with replacing missing outcome data by the last observation carried forward (LOCF) method.

As a post-hoc analysis on a subset of patients with SCORAD data available for all time points, a repeated measures ANCOVA for differences to baseline of SCORAD values was used to test if changes over time were different for the two groups (time by group effect).

The nonparametric bootstrapping method was used to generate a picture of variability around the arithmetic mean for the cost-effectiveness analyses. The original sample was bootstrapped 1000 times in order to obtain 1000 means for costs and effect differences. Each bootstrap sample was adjusted for confounding variables as previously described.

For detailed description and sample size calculation, see the previously published article [16]. Statistical analyses were performed according to a predefined statistical analysis plan using PASW Statistics 18.0 (SPSS Chicago, IL) and SAS for Windows, version 9.2 (SAS Institute, Cary, NC, USA).

## Results

### Population

**Figure 2:**
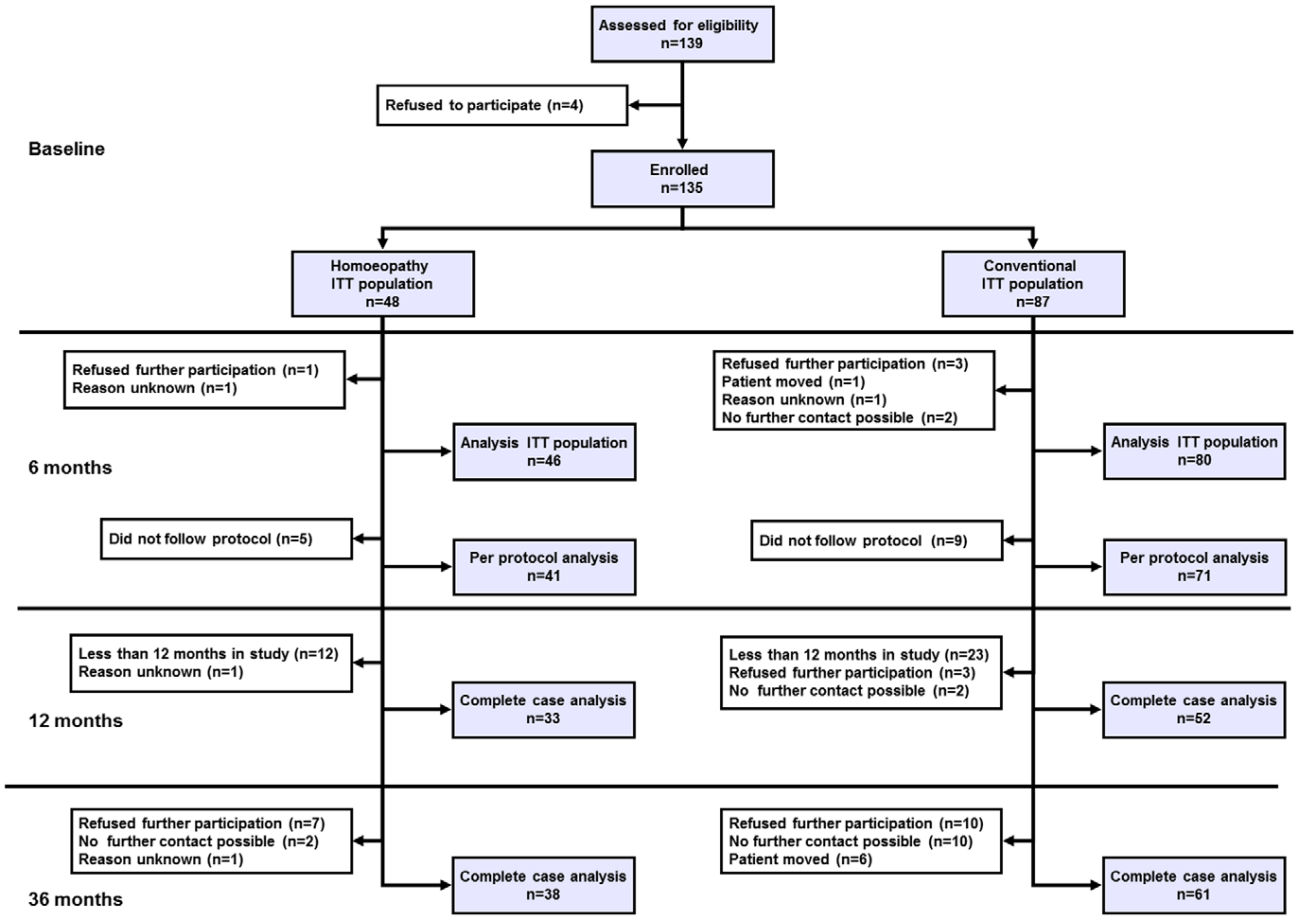
Trial flow chart (ITT: intention to treat).

135 children were included into the study and analyzed in the primary analysis after 6 months (mean age 4.01±2.97 (SD), 48% girls, conventional group n = 87, homeopathic group n = 48, Table 1). Children were recruited by 26 physicians experienced in the treatment of atopic eczema in children (10 homoeopaths and 16 conventional doctors). For details on the doctors’ specialisations please see the previously published article [16]. After 36 months, data from 99 participants (38 in the homeopathic and 61 in the conventional group) were available (Fig. 2). Reasons for missing follow-up data included refusal of further participation, relocation, or not contactable.

**Table 1:** Baseline characteristics of study participants.

	Homoeopathy			Conventional			
	n	mean±SD or n (%)	Median	n	mean±SD or n (%)	Median	P-value
**PATIENTS**							
**Age (years)**	48	4.3±2.9	4.0	87	3.9±3.0	3.0	0.300
**Gender female**	48	26 (54.2%)		87	39 (44.8%)		0.369
**Siblings**	47	1.1±1.0	1.0	84	1.0±1.2	1.0	0.530
**Symptom duration (years)**	48	3.5±2.6	3.0	86	2.9±2.7	2.0	0.057
**Treatment duration (years)**	46	3.0±2.7	2.0	83	2.7±2.9	1.0	0.364
**Additional atopic disease**							
** Allergic rhinitis**	47	4 (8.5%)		84	8 (9.5%)		1.000
** Allergic asthma**	47	2 (4.3%)		84	8 (9.5%)		0.330
** Food allergy**	47	10 (21.3%)		84	10 (11.9%)		0.205
** Other**	47	6 (12.8%)		84	8 (9.5%)		0.568
**Allergy test**	48	21 (43.8%)		87	36 (41.4%)		0.856
**Allergen positive**	19	11 (57.9%)		30	14 (46.7%)		0.561
**Three item severity score (TIS) (0–9)** a	48	3.8±1.6	3.0	87	4.5±1.5	4.0	0.010
**Therapy for AE during previous 12 months**							
** Basic skin care**	47	40 (85.1%)		84	73 (86.9%)		0.795
** Avoidance of certain food**	47	25 (53.2%)		84	33 (39.3%)		0.144
** Alternative therapies**	47	19 (40.4%)		84	3 (3.6%)		<0.001
**Drug therapy**	47	42 (89.4%)		84	71 (84.5%)		0.598
**Number of different medications**	47	2.2±1.7	2.0	84	2.3±1.9	2.0	0.852
**Corticosteroids**	47	12 (25.5%)		84	40 (47.6%)		0.016
**Topical calcineurin inhibitors**	47	18 (38.3%)		84	26 (31.0%)		0.443
**Antihistamines**	47	9 (19.1%)		84	18 (21.4%)		0.825
**SCORAD Total score** b	48	31.3±14.1	30.9	87	22.8±13.4	19.9	0.001
**SCORAD Extent**	48	18.7±19.4	13.3	87	12.9±16.0	7.0	0.066
**SCORAD Intensity**	48	6.1±2.7	6.5	87	4.1±2.6	3.0	<0.001
**SCORAD Subjective symptoms**	48	6.1±4.6	5.0	87	5.9±5.0	5.0	0.856
**Children’s QoL CDLQI** c	28	3.8±2.6	3.5	49	4.8±3.6	4.0	0.189
**ACCOMPANYING PARENT**							
**Age (years)**	47	36.7±6.1	36.0	84	32.7±6.3	32.5	<0.001
**Gender female**	47	41 (87.2%)		84	76 (90.5%)		0.568
**Single parent**	47	7 (14.9%)		84	21 (25.0%)		0.192
**Education**	47			84			<0.001
** A-level**		31 (66.0%)			22 (26.2%)		
** University, College**		20 (42.6%)			13 (15.5%)		
**Net income per month**	38			61			<0.001
** <2000 Euro**		16 (42.1%)			41 (67.2%)		
** 2000–4000 Euro**		18 (47.4%)			20 (32.8%)		
** >4000 Euro**		4 (10.5%)			0 (0.0%)		
**Social class**	47			84			<0.001
** Low**		2 (4.3%)			29 (34.5%)		
** Middle**		17 (36.2%)			34 (40.5%)		
** High**		28 (59.6%)			21 (25.0%)		
**Parents’ QoL** d							
** Psychosomatic wellbeing**	47	65.0±20.9	69.4	84	64.4±21.3	69.4	0.890
** Effects on social life**	47	87.2±16.7	91.7	84	84.6±15.4	87.5	0.384
** Confidence in medical treatment**	45	62.8±21.6	60.0	84	67.4±18.1	65.0	0.195
** Emotional coping**	47	72.9±19.3	75.0	84	71.7±21.5	75.0	0.762
** Acceptance of the disease**	46	70.4±22.1	75.0	84	68.6±24.4	75.0	0.682
**Expected symptom improvement (0–6)** e	46	4.4±1.2	4.0	83	3.8±1.2	4.0	0.013
**Costs during 12 months before study [in EURO]**		**mean±SD**	**95% CI**		**mean±SD**	**95% CI**	
**Medication**	48	119.5±146.5	77.0;162.1	87	109.6±150.7	77.5;141.7	0.493
**Hospital**	48	22.6±156.2	−22.8;67.9	87	12.4±81.6	−5.0;29.8	0.950
**Physician contact**	48	86.6±68.0	66.9;106.4	87	102.3±95.0	82.1;122.6	0.190
**Medical aids and adjuvant therapies**	48	254.6±563.2	91.0;418.1	87	113.5±264.2	57.1;169.8	0.032
**Indirect costs**	48	134.0±460.9	0.2;267.9	87	32.2±105.1	9.8;54.5	0.527
**Total**	48	617.3±841.2	373.0;861.6	87	370.0±380.8	288.8;451.1	0.164

a high score = high intensity;

b <25 mild, 25–50 moderate, >50 severe disease;

c high score = low QoL (only children of age 3 to 16 could be questioned, because CDLQI was only validated for that age group);

d high score = high QoL;

e 0 = not sure, 6 = very sure;

AE: atopic eczema, QoL: quality of life, SD: standard deviation, CI: confidence interval.

Patient preferences resulted in the following baseline differences: patients in the homeopathic group showed more severe SCORAD scores and a trend to a longer symptom duration, while the TIS score was higher in the conventional group. On average, the parents of the homoeopathic group were older, had a higher income level and were better educated, i.e. had a higher social status than the parents of the conventional group (Table 1). In addition, higher baseline costs in the homoeopathic group were seen. These differences in baseline characteristics were similar for the patients who were still available to be assessed at 36 months.

### Outcome Parameters

**Figure 3:**
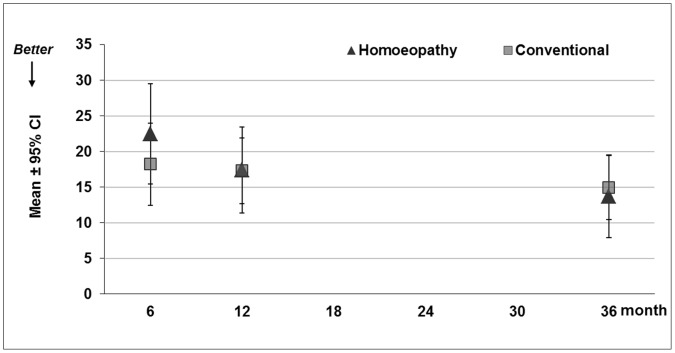
SCORAD at 6, 12 and 36 months, adjusted mean ±95% confidence interval (CI) per group from multilevel models (ANCOVA) with fixed effects age, gender, baseline value, TIS-score, social status, expectation of the parents, and random effect physician (lower values indicate lower disease severity).

After 36 months the primary outcome parameter SCORAD showed no significant differences between groups: homeopathy group 13.68±2.91 (adjusted mean±SE) 95% CI [7.88–19.48] vs. conventional group 14.90±2.25 [10.41–19.40], p = 0.741) (Table 2, Fig. 3). When replacing missing SCORAD values at 36 months by LOCF as a sensitivity analysis, results remained similar (homeopathy group 13.29±2.51 [8.30–18.28] vs. conventional group 15.24±1.92 [11.43–19.05], p = 0.541). Neither the SCORAD at six or 12 months [29] nor the SCORAD subscales showed significant differences (Table 2; and Witt et.al [16]).

**Table 2:** Intention to treat analyses of SCORAD and secondary outcomes at 36 months (adjusted means or proportions and confidence intervals (CI) from multilevel models (ANCOVA or GEE) with fixed effects age, gender, baseline value, TIS-score, social status, expectation of the parents, and random effect physician).

	Homoeopathyn = 37		Conventionaln = 61		
	Mean	95% CI	Mean	95% CI	P-value
PATIENTS					
SCORAD Total score^a^	13.7	7.9–19.5	14.9	10.4–19.4	0.741
SCORAD Extent	5.0	2.1–7.8	3.4	1.1–5.8	0.406
SCORAD Intensity	3.1	1.9–4.4	3.4	2.4–4.3	0.777
SCORAD Subjective symptoms	2.0	0.8–3.2	2.3	1.4–3.3	0.682
Children’s QoL CDLQI (0–30)^b^	2.2	1.4–3.4	1.8	1.2–2.8	0.627
Different medications per patient	0.7	0.4–1.4	0.7	0.5–0.9	0.904
Other physician visits	1.7	0.4–3.0	1.5	0.5–2.4	0.754
**MEDICATION (31–36 months)**	Proportion of patients (%)	95% CI	Proportion of patients (%)	95% CI	P-value
Corticosteroids	9.5	2.9–27.1	10.2	3.8–24.8	0.889
Topical calcineurin inhibitors	n.c.	n.c.	n.c.	n.c.	n.c.
Antihistamines	1.3	0.2–10.9	4.0	1.7–9.1	0.304
Basic skin care	66.9	53.0–78.4	61.0	50.0–70.9	0.557
Still treated by study doctor	71.7	47.6–87.6	62.9	50.1–74.0	0.5068
ACCOMPANYING PARENT	Mean	95% CI	Mean	95% CI	P-value
Parents’ QoL^c^					
Psychosomatic wellbeing	82.4	76.8–88.0	77.1	73.0–81.1	0.136
Effects on social life	94.8	91.8–97.9	94.1	91.8–96.5	0.726
Confidence in medical treatment	77.7	71.1–84.3	77.5	72.7–82.4	0.959
Emotional coping	87.0	81.1–92.8	85.0	80.8–89.2	0.597
Acceptance of the disease	83.3	76.0–90.6	84.0	78.4–89.6	0.869
COSTS (31–36 months)					
Medication	36.0	7.3–64.7	36.7	14.4–59.1	0.969
Hospital	n.c.	n.c.	n.c.	n.c.	n.c.
Physician contact	119.8	95.1–144.5	45.4	27.5–63.4	<0.001
Study doctor	83.8	63.8–103.9	17.1	0.8–33.5	<0.001
Other physicians	35.1	12.8–57.5	26.6	10.3–43.0	0.546
Medical aids and adjuvant therapies	53.0	18.6–87.3	16.1	0.0–41.0	0.093
Indirect costs	9.3	0.0–22.1	1.9	0–11.7	0.369
Total costs	217.0	154.1–279.9	99.9	53.7–146.1	0.005

a <25: mild, 25–50: moderate, >50: severe disease.

b higher scores refer to lower QoL.

c higher scores refer to higher QoL.

SCORAD: Scoring atopic dermatitis; CDLQI: Children Dermatology Life Quality Index; QoL: quality of life; CI: confidence interval, n.c.: not computable.

**Figure 4:**
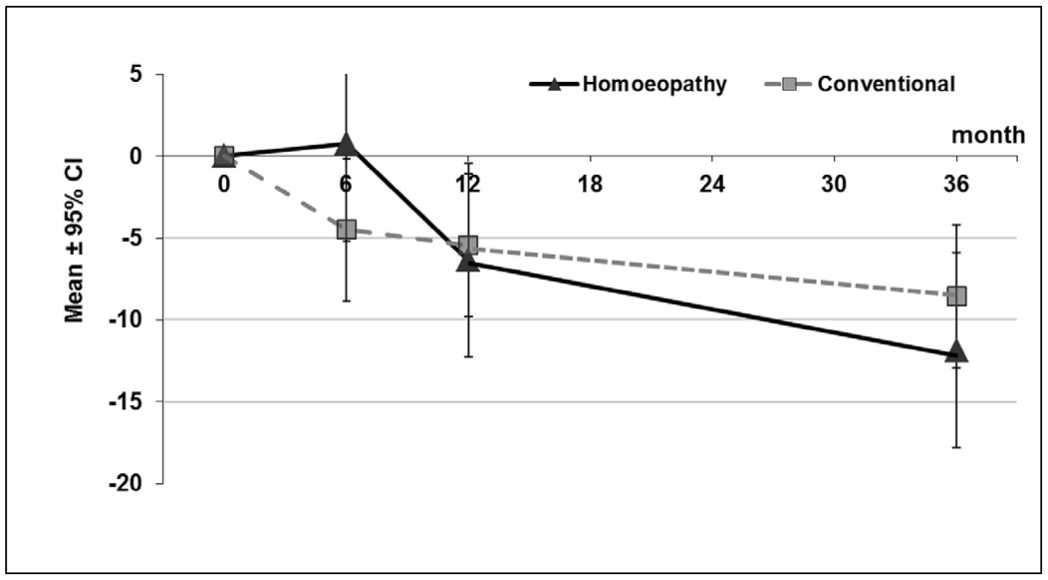
SCORAD differences to baseline at 6, 12, and 36 months, adjusted mean ±95% confidence interval (CI) per group from repeated measures multilevel model with time and time-by-group interaction and fixed effects age, gender, baseline value, TIS-score, social status, expectation of the parents, and random effect physician; post-hoc analysis on complete cases (patients with SCORAD data available for all time points); lower mean values indicate greater improvement.

No significant overall group effect was found between homoeopathy and conventional treatment in the repeated measures analysis when analyzing SCORAD differences to baseline for the subset of patients with SCORAD data available for all time points (p = 0.908). However, SCORAD values in general decreased significantly over time (p<0.001). This change in time seemed different for the two groups with a faster improvement in the conventional treatment group at 6 months, and a catching up of the homoeopathy group after 12 months (p = 0.055, Fig. 4).

No significant differences could be observed at any time point for adjusted response rates based on SCORAD values. At 36 months a 33% response (defined as an improvement of at least 33% in the SCORAD) of 63.9% was seen in the homoeopathic and 64.5% in the conventional group (p = 0.949). A 50% response was seen at 36 months in 52.0% of patients in the homoeopathic and 52.3% of patients in the conventional group (p = 0.974).

At 36 months the quality of life of the children and parents was similar in both groups in the short and long-term (Table 2).

### Conventional Treatment

At 36 months the frequency of daily basic skin care was reduced compared to baseline, and comparable in both groups, as was the number of different medications (including corticosteroids and antihistamines) according to the patients’ documentation (Table 2). One patient (2.6%) in the homoeopathic group and one patient (1.6%) in the conventional group took corticosteroids of the weak group (group I according [19]), while one patient (1.6%) in the in the conventional group took moderately potent corticosteroids (group II). Three patients (7.9%) in the homoeopathic and 11 patients (18.0%) in the conventional group took potent corticosteroids (group III). Details of the homeopathic treatment within the first 12 months of the study have previously been described [16].

### Subgroups

Considering only patients still treated by the study doctor they have chosen at study start at 36 months (n = 62), SCORAD values showed no differences between the groups (homeopathic 14.28±3.46, 95% CI [7.36–21.21] vs. conventional group 15.89±2.72 [10.46–21.33], p = 0.722). Similar results were found for patients no longer receiving study therapy at 36 months (n = 33) (homeopathic 12.26±4.17 [3.92–20.60] vs. conventional group 14.33±3.09 [8.16–20.50], p = 0.686). A test of interaction (effect modification) from still receiving study therapy was not significant (p = 0.937).

### Adverse Events

The number of patients reporting adverse events at 36 months was similar in both groups. Two patients (5.3%) in the homoeopathy group and five (8.2%) in the conventional group reported adverse events (p = 0.704). The following eight adverse events were reported in the homoeopathy group: pruritus (n = 2), burning sensation (1), reddening (1), dry skin/flaky skin (2), burns (1), herpes zoster (1); and 12 adverse events in the conventional group: pruritus (3), burning sensation (3), reddening (2), dry skin/flaky skin (3), and allergic reaction (1). The number of adverse events reported was also similar in the two groups at six and 12 months, with one child in the conventional group needing hospitalisation due of a worsening of the atopic eczema with additional streptococcal infection [16].

### Economic Analyses

Economic data from the patients’ questionnaires were available for 135 patients at baseline and for 98 patients for the study period from 31 to 36 months. During the 12 months before baseline costs were 617.28 Euro 95%CI [373.02–861.55] in the homoeopathic group and 369.96 Euro [288.80–451.13] in the conventional group (p = 0.164, Table 1).

### Cost-comparison analyses

Significant total cost differences were found at the long-term follow-up from 31 to 36 months after baseline (homoeopathic group: 216.99 Euro [154.12–279.87]; conventional group: 99.93 Euro [53.75–146.12], p = 0.004; Table 2). The respective physician’s contacts were one of the main cost drivers (homoeopathic group: Euro 119.77 [95.06–144.49]; conventional group: Euro 45.44 [27.48–63.41], p<0.001), particularly contacts with the study doctor (homoeopathic group: 83.84 Euro [63.84–103.85]; conventional group: 17.12 Euro [0.75–33.49], p<0.001). In the sensitivity analysis the total costs were 183.17 Euro [119.65–246.69] in the homoeopathic, and 100.18 Euro [53.19–147.17] in the conventional group (p = 0.043).

## Discussion

In this observational comparative effectiveness study, no significant long-term outcome differences were seen after 36 months in children with atopic eczema when treated conventionally compared to homoeopathic treatment; neither were short-term differences seen (at six or 12 months [16]). Both groups substantially improved during the observation period and in both groups every tenth patient used corticoids. Patients in the conventional groups showed a trend towards earlier improvements. Costs, however, were higher in the homoeopathic group.

The design of the study (observational, usual-care and multicentre setting) allows evaluation of a therapy’s comparative effectiveness considering the patients’ own preferences and therapy choices. In our study the treatment was individualised and to reflect a more realistic care additional medication was not forbidden. Data collection was performed using a variety of sources including the affected child, their parents, study doctors and external blinded raters, to improve the objectivity and validity of the study outcomes.

The aim of this study was to reflect the real world situation and to compare conventional and homeopathic care provided by physicians in a usual care setting. Thus, we chose to take patients’ and/or parents’ therapy preferences into account, making randomisation not possible. The observational design resulted in relevant baseline differences between the two groups. In the homoeopathic group severity of disease appeared higher compared to the conventional group. Eczema conditions according to the SCORAD were less favourable (especially the intensity and extent of atopic eczema) and the disease duration was longer. In addition, parents in the homoeopathic group were older, had a higher social class background and a higher treatment expectation. To take baseline differences into account, we adjusted our analyses for these factors. However, it is possible that other unknown and unmeasured factors might have influenced the results. If other confounding factors were present but not accounted for, or if the performed adjustments were not sufficient (e.g. due to broad value categories or measurement error), residual confounding might be present. If adjustments did not sufficiently balance disease severity, then results might be biased in favour of either the conventional or the homoeopathic group. Therefore, the non-randomised design is a clear limitation of our study regarding the internal validity of our results [16].

At six, 12 and 36 months SCORAD severity was comparable in both groups, although patients in the conventional group took more conventional medication in the first year, e.g. corticosteroids, antihistamines or pimecrolimus and tacrolimus [16]. Baseline use of pimecrolimus and tacrolimus was comparable in both groups, in contrast to less use of corticosteroids in the homoeopathic group than in the conventional group. While at six months, the use of these anti-inflammatory drugs was lower in the homoeopathic group, after 36 months around ten percent of patients in both groups used corticoids. Overall medication use decreased during the trial in both groups compared to baseline values. In both groups the frequency of basic skin care was comparable. However when interpreting these results, one should also take into account that atopic eczema can improve spontaneously in young children.

Although disease specific costs in both groups appear lower in the third year compared to the first year, the long-term costs were more than twice as high in the homeopathy group compared to the conventional group. Costs were mainly driven by doctors’ fees and paying for medical aids. As described in the methods section, the mean costs during months 7–12 of the study were used to estimate the costs for the outpatient contact at the long-term follow-up for the main analysis. We chose this method as it also seems to be a conservative approach, in assuming that the intensity of medical contact (especially for homeopathic treatment) is much higher during the first year of treatment, and that the use of these costs for estimating follow-up expenditures might lead to an overestimation of costs. Data from another prospective observational study that included 3981 patients with different diagnoses showed that children with atopic eczema [18] visited their homeopathic doctor between month 7–12 40% more often (1.5±1,7 times) than a year later (0.9±1.2 times in months 19–24). These findings were used as the basis for a sensitivity analysis, resulting still in higher costs in the homoeopathic group.

The substantial and statistically significant cost-differences between the groups found during the first year of treatment were stable over time. The follow-up between 31 and 36 months lead to a comparable result compared to the analysis in the first year. While interpreting these differences, potential limitations should be kept in mind, particularly in regard to how the outpatient costs were estimated. Within the 31–36 months follow-up detailed therapeutic documentation was not available. However, the method of calculating the outpatient costs during the first year of the study was based on this kind of documentation.

### Conclusion

In this long-term observational study after three years, while unable to rule out residual confounding but taking patient preferences into account, treatment at homoeopathic doctors was similar, yet not superior to treatment at conventional doctors for children with mild to moderate atopic eczema, but still had higher costs.
